# STR typing from punched FFPE tissues: resolving suspected contamination and fetal–maternal mixture in two cases

**DOI:** 10.1007/s00414-026-03891-1

**Published:** 2026-06-25

**Authors:** Barbara Di Stefano, Rossana Bussani, Solange Sorçaburu Ciglieri, Maurizio Pinamonti, Claudia Biagi, Manuela Schneider, Fabrizio Zanconati, Paolo Fattorini

**Affiliations:** Department of Medicine, Surgery and Health, University of Trieste, Trieste, 34149 Italy

**Keywords:** FFPE, Punched samples, STR typing

## Abstract

The analysis of mixed or contaminated biological materials represents a persistent challenge in both forensic genetics and diagnostic pathology. This study presents two casework applications demonstrating the analytical value of a targeted FFPE punching strategy coupled with STR genotyping for resolving complex scenarios. In the first case, an unexpected focus of high-grade urothelial carcinoma within a prostatic surgical specimen raised concerns regarding cross-sample contamination. STR profiling of microdissected punches—respectively containing the carcinoma and benign prostatic tissue—yielded fully concordant single-source profiles (LR = 2.5 × 10²¹), thereby unequivocally establishing a common donor origin and excluding inadvertent tissue transfer. In the second case, involving paternity assessment of mechanically aborted embryonic material from an alleged sexual assault, morphologically guided punching enabled selective isolation of chorionic villi from a grossly heterogeneous specimen. Although low-level maternal DNA was detected, mixture interpretation allowed reconstruction of a complete embryonic genotype, resulting in a conclusive paternity (LR = 2.2 × 10¹⁰). Altogether, these observations underscore the analytical robustness, cost-efficiency, and operational simplicity of punched-FFPE-based STR typing as a first-line approach for contamination assessment and targeted genotype recovery.

## Introduction

Mixed or contaminated biological samples present significant challenges in forensic and clinical laboratory. These samples contain genetic material from multiple individuals, with mixed samples typically arising from crime scenes where biological traces from different contributors are deposited simultaneously [1, 2]. Conversely, contamination can occur during collection, storage, or processing, introducing exogenous tissues or cells [3, 4].

Mixed liquid samples are common findings in several forensic medicine scenarios. For example, in rape cases, DNA analysis of vaginal swabs often reveals a mixed profile—with variable proportions—of the victim and the perpetrator. In such cases, although identification and separation of sperm cells (if present) is preferable, the interpretation of genetic testing remains challenging [5–7]. For this reason, several in silico systems have been developed in recent years to calculate the Likelihood Ratio (LR) that the suspect is one of the biological contributors to the DNA mixture. In other cases, such as aborted fetal material, the sample may consist of a mixture of fetal and maternal tissue and blood [8]. To overcome the challenges of result interpretation, microscopic assessment of the sample and selection of fetal tissues guided by laser microdissection can be performed [9].

Solid contamination in pathology laboratories is a critical threat to the accuracy of histopathologic diagnosis. Contamination sources span the entire workflow—from grossing and embedding to microtomy and staining—with risks arising from cross-sample carryover. Tissue “floaters” and cross-contaminants have been documented in stainer baths and transport fluids (particularly for urothelium, colorectal adenocarcinoma, and placental villi), leading to potential diagnostic errors [10–12].

In recent years, the use of punched FFPE samples—small cylindrical cores obtained from archived tissue blocks—has gained attention as a practical approach for high-throughput molecular studies, such as tumor genotyping for personalized therapies [13–14]. This method allows researchers to selectively sample regions of interest, avoiding undesirable tissues of the block (being necrotic or containing blood) [15–16]. In addition, this procedure is inexpensive and rapid without exhausting entire blocks, and thereby preserving valuable archival material.

In the present work, we describe the use of the punching procedure coupled with STR (Short Tandem Repeat) typing, in two cases. The first involves a suspected contamination in a prostatic Thulium Laser enucleation. The second concerns a paternity test conducted on aborted fetal material. In both cases, the punching procedure proved to be an irreplaceable tool for accurate, inexpensive and time-saving molecular diagnosis.

## Material and methos

The two cases and the methods used are described below.

### Case 1

A 70-year-old patient underwent prostatic Thulium Laser enucleation with clinical diagnosis of prostatic hyperplasia.

**Fig. 1:**
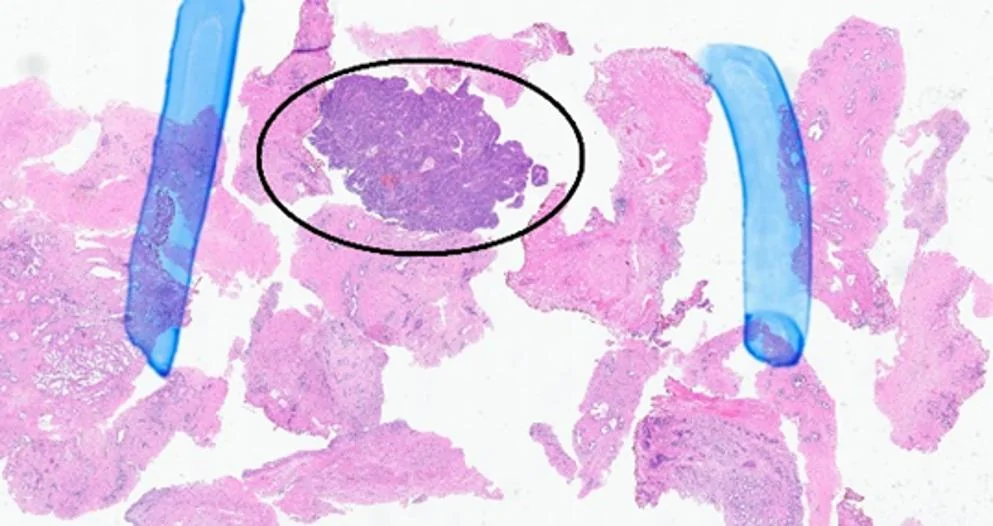
Microscopical examination (2.5 x) of the prostatic sample. The urothelial carcinoma -of darker color- is shown within the ellipses (**a**)

During histological analysis, including numerous prostatic fragments with adenofibroleiomyomatous hyperplasia, chronic inflammation, extensive squamous metaplasia, and reactive changes, two small fragments were also identified, representing high-grade urothelial carcinoma (G2), likely originating from the prostatic urethra (see Fig. 1). Standard ISO procedures [17] were followed for the entire workflow.

Given the absence of clinical data regarding the possible presence of a urothelial neoplasm, either past or present, artifactual transport of material from another sample was taken in to consideration [10, 18].

Two regions of the FFPE sample were then selected for STR profiling: one containing the carcinoma (sample 1_C) and the other containing benign lesions (1_A), as shown in Fig. 1.

### Case 2

An 18-year-old girl underwent an abortion at approximately eight weeks of gestation. Importantly, the conception was believed to have occurred as a result of sexual assault. Vacuum aspiration followed by curettage was performed. After removal, the two specimens were placed in 50-ml Falcon tubes and stored at − 20 °C. The paternity of the aborted embryo was established nine months later, per instructions of the judge.

**Fig. 2:**
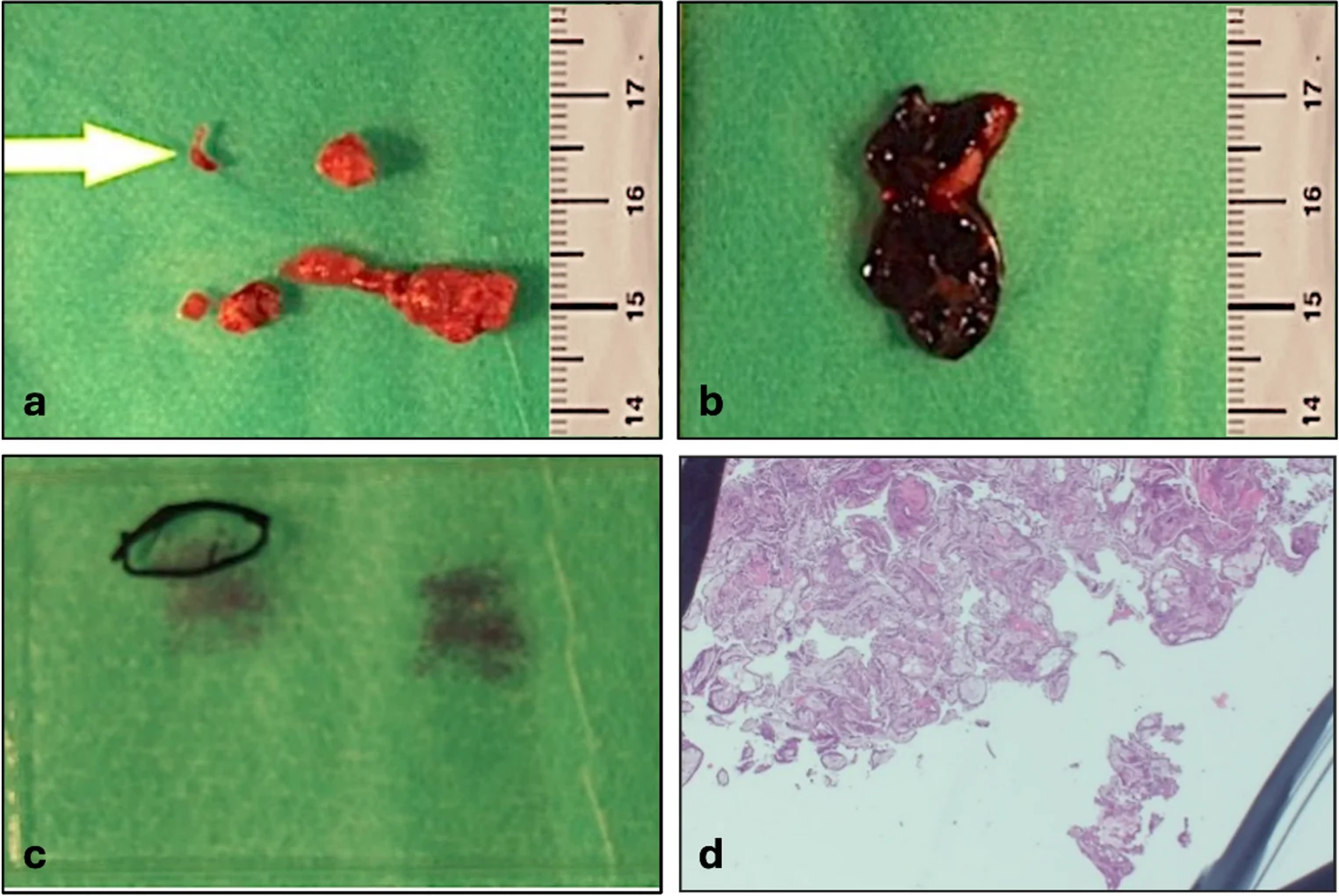
Macroscopic and microscopic examination of the aborted material. (**a**) Sample obtained by vacuum aspiration. The arrow indicates a fragment suspected to represent chorionic villi. (**b**) Sample obtained by curettage. (**c**) Portion of the slide 2_V with the circled area selected for further analysis. (**d**) Histological examination of the selected area (20 x)

The aborted material was briefly washed twice in physiological solution at 4 °C and then examined. Several necrotic and hemorrhagic areas were macroscopically evident in the sample obtained by vacuum aspiration (Fig. 2).

After macroscopic identification of a fragment likely representing chorionic villi, this specimen was fixed for 12 h and then processed according to standard ISO procedures [17]. The resulting slide was stained with H&E and examined microscopically. As part of this sample was actually represented by chorionic villi (see Fig. 2), the identified specimen was named sample 2_V.

As reference samples, we were provided with saliva swabs from both the mother (sample 2_M) and the alleged father (that is the suspect; sample 2_S).

### Punched FFPE samples

After microscopic examination, the areas of interest were marked on the slide for both Case 1 and Case 2 (see Figs. 1 and 2c). The annotated H&E slide was then used as a guide to select the corresponding region from the FFPE block. A 3-mm biopsy punch (Kai Medical, Japan) was used to penetrate approximately 1 mm into the FFPE tissue block to facilitate sampling and ensure proper alignment for subsequent core recovery [14].

After chilling the FFPE block at − 20 °C for half an hour, the block was sectioned at 10 μm thickness. Eight core sections were retrieved with a sterile needle during sectioning and transferred into sterile 1.5-ml Eppendorf tubes.

Before processing each block, the microtome blade was sterilized with a 0.5% bleach solution, followed by two washes with 90% ethanol. All other recommended procedures to prevent contamination were strictly followed [3, 4].

### DNA extraction and typing

To remove any accidental surface contamination, the core sections were washed for 10 s in a 0.5 ml of 0.5% bleach solution. After centrifugation at full speed for 1 min, the samples were washed twice with 1 ml of sterile bi-distilled water. They were then dried for 10 min at 56 °C under vacuum. After deparaffinization with xylene [19], the core sections were subjected to DNA extraction following standard procedures previously described [19]. The final elution volume was 30 µl. Extraction negative control (ENC) was processed in parallel.

DNA quantification was performed by qPCR using the PowerQuant kit (Promega) under standard conditions [20].

Multiplex STR amplification was carried out using the PowerPlex ESI kit (Promega) under standard conditions, with 0.8 ng of template DNA (as determined by qPCR) and 30 PCR cycles [21]. DNA typing was performed on a SeqStudio genetic analyzer (Applied Biosystems) using ID XR Software v1.6 (Applied Biosystems). No template control (NTC) and the ENC were included in each run.

All recommended procedures to prevent contamination were strictly followed [3, 4, 22].

### Data analysis

To assess the weight of the genetic typing, forensic parameters were computed using standard methods. Specifically, the Likelyhood Ratio (LR, that is 1/RMP; Random Match Probability) for Case 1 was calculated with EuroForMix (version 4.0.8) [23]. Familias (version 3.2.9) [24, 25] was used to compute the probability of paternity for Case 2. Suitable population genetic database were applied in both analyses.

EuroForMix was also used to estimate the degradation level of the three FFPE samples. Finally, allele peak areas obtained from ID XR Software v1.6 were exported and used as input for calculations performed in Excel.

## Results and discussion

The results of DNA quantification are reported in Table 1. High DNA yields were obtained from the three punched samples. The Auto/Deg ratio showed higher values in the samples from Case 1, indicating a greater degree of degradation. It is likely that the lower level of degradation observed in sample 2_V is due to its shorter fixation time (12 h) instead of the 48 h of samples from case 1 [26]. No PCR inhibition was detected, as indicated by the Cq values of the IPC (Internal Positive Control) target.

**Table 1:** Molecular data of the three FFPE samples studied here. Auto, Deg, Y refer to the data of the qPCR quantification of the Autosomal, Degradation and, respectively, Y target of the PoweQuant kit (ng/µl). IPC: internal Positive Control of the PowerQuant (in Cq). Deg. Slope: Degradation Slop as assessed by the Euroformix software

Case	Sample	Auto	Deg	Y	IPC	Auto/Deg	Deg. slope
1	1_C	0.184	0.031	0.072	20.8	5.93	0.489 ± 0.05
	1_A	4.931	0.872	3.038	20.8	5.65	0.490 ± 0.06
2	2_V	0.382	0.195	n.d.	20.9	1.95	0.611 ± 0.07

All DNA samples underwent standard STR typing. Neither the ENC nor the NTC produced amplicons. The genetic findings can be summarized as follows.

### Case 1

Both samples 1_C and 1_A produced complete male profiles, with degradation slopes of 0.489 and 0.490, respectively, consistent with the Auto/Deg ratios obtained using the PowerQuant kit (the undegraded positive control sample showed a slope of 0.987).

All 16 STR loci included in the PowerPlex ESI kit were successfully amplified generating two single donor profiles. Full concordance was observed between the two samples. The LR was approximately 2.5 × 10²¹, indicating that the small tissue inclusion originated from the same donor from whom the entire sample was taken. Therefore, the possibility of contamination was excluded.

### Case 2

**Fig. 3:**
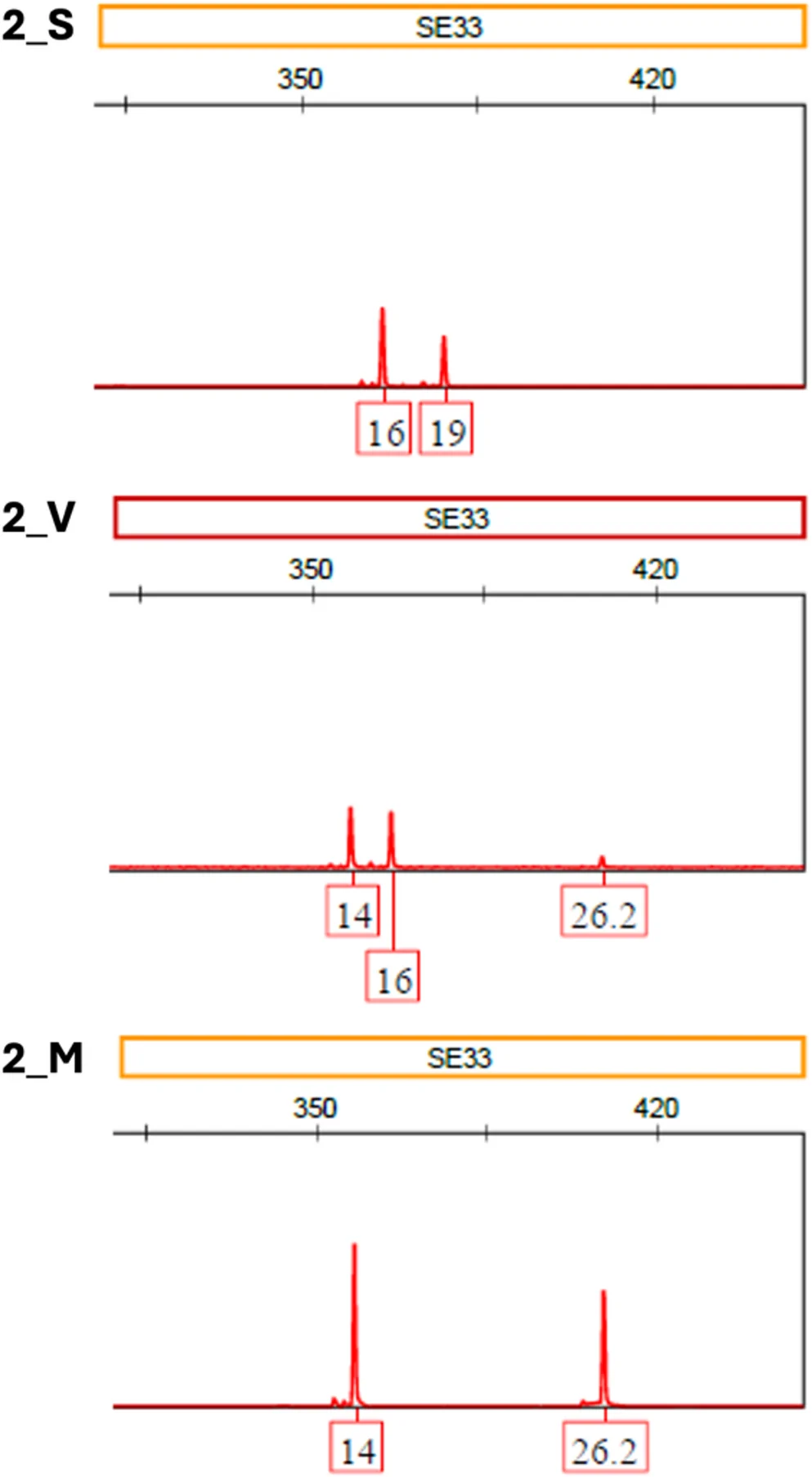
STR typing of the SE33 locus of the PowerPlex ESI kit. The punched sample from the aborted material (2_V) shows minimal traces of maternal DNA (allele 26.2 of sample 2_M). Sample 2_S: saliva swab of the suspect

Sample 2_V yielded a complete female profile with a degradation slope of 0.611. Electropherogram analysis revealed that 11 of the 16 STR loci displayed additional alleles attributable to contamination by maternal DNA, as confirmed by comparison with the maternal reference saliva profile (sample 2_M). The peak areas of these additional alleles, expressed as a percentage of the sum of the main allele peak areas, ranged from 7.1% (marker D1S1656) to 23.0% (marker D22S1045) (see also Fig. 3). Using the mixture analysis tool in EuroForMix, the proportion of maternal DNA in the profile was estimated to be, on average, 18% (± 5.1%).

Thanks to the availability of the maternal profile, a single profile was extrapolated from sample 2_V and then used for comparison with sample 2_S. The resulting LR was 2.2 × 10¹⁰, corresponding to a probability of paternity greater than 99.99999999%, thus allowing a conclusive identification of the biological father (and of identity suspect).

## Conclusions

In this paper, we describe the successful use of punched FFPE samples for STR typing in two independent cases.

In the first case, contamination was suspected in a FFPE block derived from prostate surgery. This rare yet potentially serious event was excluded because the genetic profile obtained from the questioned tissue punch matched the patient’s genotype. In case of contamination, in fact, such exogenous sample would give a different profile [1]. Of great relevance, this finding led the clinicians to suitable therapeutic protocols and any kind of medical liability was avoided.

In Case 2, the punched-FFPE approach enabled accurate selection of embryonic tissue (chorionic villi) within a mechanically aborted sample. Genetic typing revealed the presence of maternal contamination; however, this did not prevent the recovery of the embryonic genotype. As a single, unambiguous genetic profile of the embryo was obtained, the challenging issue of mixed-sample interpretation [27, 28] was avoided during the paternity calculations.

Definitive STR typing results were achieved using the simple and inexpensive punched-FFPE method. We believe that the two cases described here represent paradigmatic examples of the effective integration between the expertise of the Molecular Pathology laboratory and that of the Forensic Genetics laboratory. We believe that punched-driven STR typing could be included in routine practice whenever exogenous contamination is suspected during histopathological examination. Similarly, the punched-FFPE method should be considered the first-line approach for paternity testing of aborted embryos due to its simplicity and the minimal laboratory equipment required. As technical tip, to facilitate the collection of the selected core sample, we recommend the refrigeration of the punched FFPE before sectioning.

## Data Availability

No datasets were generated or analysed during the current study.
